# Oral Hygiene Practices and the Awareness of Perio-Systemic Interrelationship Among the Population of Ranchi City: A Hospital-Based Study

**DOI:** 10.7759/cureus.32368

**Published:** 2022-12-09

**Authors:** Vivek Gupta, Santosh K Verma, Priyanka Singh, Mohd. Sameem Alam, Bhavana Gupta, Priyanka Kumari

**Affiliations:** 1 Department of Periodontology and Oral Implantology, Dental College, Rajendra Institute of Medical Sciences (RIMS), Ranchi, IND; 2 Department of Periodontology, Dental College, Rajendra Institute of Medical Sciences (RIMS), Ranchi, IND; 3 Department of Periodontology, Jai Ram Hospital, Ghaziabad, IND; 4 Department of Periodontology, Family Dental and Medical Centre, Delhi, IND; 5 Department of Oral Pathology and Microbiology, Awadh Dental College and Hospital, Jamshedpur, IND

**Keywords:** periodontal conditions, systemic diseases, ranchi, interrelation, awareness

## Abstract

Background:The concept of the pathogenesis and etiology of periodontal disease, with their infectious and chronic natures, usually facilitates acknowledging the possibility of these infections influencing events elsewhere in the body. Concurrent awareness and recognition of the interaction between systemic and oral diseases are one of the enormous advances that require a periodontist to not only strictly direct their knowledge toward prevention and treatment but also spread awareness about the same among the unknown. Thus, the primary goal of our study was to assess public awareness of periodontal and systemic interrelationships with oral hygiene practices in Ranchi, Jharkhand.

Methodology: A total of 800 subjects between ages 18 and 60 years visiting the outpatient department of periodontology, Dental Institute, Rajendra Institute of Medical Sciences (RIMS), were randomly selected for inclusion in the study. After the oral hygiene checkup, the patients were presented with a self-constructed questionnaire form, where patients' awareness and knowledge about perio-systemic interrelationship and their patterns about oral hygiene practices were assessed.

Results:The data collected was analyzed using mean and standard deviation (SD), while the chi-square (χ^2^) test was to evaluate the mean difference. The results of our study showed a fair oral hygiene index, minimal oral hygiene practices, and a lack of awareness regarding the interrelationship between bad oral health and systemic diseases among the population of Ranchi. Out of 800 subjects, the majority (around 44.25%) visited a dentist only if and when needed, and around 80% of the population continued using their toothbrushes for more than six months. In fact, awareness regarding the perio-systemic interrelationship was only among 5.12% (3.25% ± 1.87%) of the total population.

Conclusion: Within the limitations of our study, it can be concluded that there is a need to educate the general population about the pros and cons of maintaining oral hygiene. Dental awareness, along with periodontal health care and its impact on systemic health, should be intensified through various means.

## Introduction

The interrelationship between the body and the disease of the mouth as a whole is not a new concept and has been debated for over a century. A well-known theory, "the theory of focal infection," introduced by William Hunter, connected oral infections with infection in other parts of the body, which led to the removal of many infected teeth with the hope of curing the distant disease conditions [1]. It was in the 1980s and 1990s that a concerted effort at gathering scientific evidence related to the topic was made. Oral health thus became an inseparable part of general health, with a number of things influencing it, namely, oral hygiene practices, diet, socioeconomic status, literacy, and even geographic location [2].

Periodontal diseases are usually referred to as the inflammatory processes occurring in and around the tissues surrounding the teeth in response to bacterial or dental plaque accumulation. This accretion of bacteria is responsible for various chronic inflammatory responses, including the long-term release of cytokines in systemic circulation. Numerous documents have been presented in the literature proving the multifactorial nature of the diseases, incorporating microbial challenges, an immune-inflammatory response of the host, and various genetic and local factors. Cancer treatment, pregnancy, cigarette smoking, anti-epileptic drugs, systemic diseases, steroidal medications, crooked teeth, ill-fitting dentures, and oral contraceptives all raise the risk of periodontal disease [3]. Aside from the chronic state, it has been linked to increased morbidity and mortality in a variety of medical conditions, including stroke, cardiovascular disease, acquired respiratory infections, adverse pregnancy outcomes, and poor glycemic control, all of which are likely to exacerbate periodontal disease [4].

Oral health knowledge is regarded as an essential prerequisite for an individual's health-related behaviors, including their attitude toward dental care and dentists, who in turn help determine the oral health of the population [5]. It is therefore the physician's responsibility to identify chronic periodontitis in patients who present with chronic medical conditions in order to make an immediate referral for an appropriate diagnosis and treatment plan. Not only that but also any disease can be prevented by raising awareness about it, which is typically enhanced by patient education and motivation. Periodontitis and its systemic impact must be made widely known to the general public in order to aid in its management. As a result, the primary goal of our study was to assess the general population of Ranchi's awareness and knowledge of periodontal and systemic interrelationships, as well as oral hygiene practices.

## Materials and methods

A cross-sectional study was performed on 800 subjects between 18 and 60 years of age who visited the outpatient department of periodontology, Dental Institute, Rajendra Institute of Medical Sciences (RIMS), Jharkhand, for a dental checkup. Ethical clearance was obtained from the Dental Institute with IRB number RIMS/IEC/71. The subjects with a maximum of 10 scorable teeth were selected, and those with mobile, grossly decayed, impacted, or root stumps were excluded from the study. The requirement of the survey was discussed with each subject, and people who were willing to fill out the form or share their information regarding it were selected. The questions were printed both in English and Hindi. The patients were examined using probe, mirror, and tweezers (PMT) and an explorer. A simplified oral hygiene index was used to assess the periodontal condition of the patients. Following their oral examination, patients were given the appropriate questionnaire form, as well as pens or pencils, to complete and return to the examiner. The subjects who were unable to write or read had the examiner himself/herself ask the pertinent questions and complete the survey form for them.

A complete set of 17 questions was included in the survey form, with two demographic questions, 10 oral hygiene-related questions, and five periodontal and systemic-related questions. The questions were predominantly associated with the general awareness of gingival and periodontal diseases, their interrelation with systemic diseases, and their visits to the dental specialist for their dental checkups. It was recorded based on the options available in the form of survey questions (including multiple-choice and Likert scales). The content validity index was used to validate the questionnaire.

The recorded data was organized and tabulated in a Microsoft Excel (Microsoft® Corp., Redmond, WA) sheet for further analysis. The data was then transferred to Statistical Package for Social Sciences (SPSS) (IBM SPSS Statistics, Armonk, NY) software (version 20.0) for analysis and comparisons. Frequencies, percentages, means, and standard deviations (SD) were used for descriptive data, while the chi-square (χ^2^) test was used to evaluate the statistical differences between the treatment outcomes. Bar diagrams were used to represent the data, and a p-value of 0.05 was used as a significant value. Survey questions were pretested and affirmed among 10 subjects, who were further barred from examining the study tests. Taking into account the response rate with a margin of error of 5%, the sample size was resolved to 664; in order to increase the quality and strength of the study, the sample was expanded to 800. It was calculated using the formula Z^2^pq/e^2^, where Z = 2.57 for a confidence level (α) of 99%.

## Results

On evaluating the results of our study, the majority of the population were found to be between ages 18 and 30 years (40.60%) followed by 31-43 years (28.26%); thus, the major respondents of the study were middle-aged people. Out of the total population, 550 were males, and 250 were females, which represented a male-dominating nature of the society where females are still made confined to household chores. The majority of the respondents were graduates (72.5%) and were very well aware of the nature of the study, while 5% of all respondents were illiterate, and 2.87% were educated only until class VIII, who required the help of the examiner for filling up the survey form (Table 1).

**Table 1:** Representing the demographic details of the study subjects SD: standard deviation; N: number

Variable		Frequencies (N)	Percentages (%)	Mean ± SD
Age (in years)	18-30	325	40.62	37.616 ± 12.671
	31-43	226	28.26	
	44-56	152	19	
	57-60	97	12.12	
Gender	Male	550	68.75	1.315 ± 0.464
	Female	250	31.25	
Education	Illiterate	40	5	3.643 ± 0.784
	Class I-VIII	23	2.87	
	Class IX-XII	139	17.37	
	Graduate	580	72.5	
	Post-graduate	18	2.25	

On evaluation, out of 800 study subjects, the majority of the population (56.87%) presented with fair oral hygiene followed by 23.62% of subjects with poor oral hygiene. In proportions of gender, females were found to have higher proportions of the fair (20.8%) and good (61.2%) oral hygiene when compared to males (18.91% and 54.91%). In cases of poor oral hygiene status, males (26.18%) were found with a higher percentage than females (18%) (Table 2).

**Table 2:** Representing the correlation of oral hygiene status with gender S: significant

Gender	Frequency/percentage			Chi-square (χ^2^)	P-value	Significance
	Good	Fair	Poor	6.3812	0.0411	S
Male (550)	302/54.91	104/18.91	144/26.18			
Female (250)	153/61.2	52/20.8	45/18			
Total	455 (56.87%)	156 (19.5%)	189 (23.62%)			

Out of 18 post-graduate, the majority (55.54%) presented with good oral hygiene status, while an equal amount had fair and poor (22.23%) oral hygiene status. Among the graduates, 52.58% of them had good oral hygiene status, which was followed by poor (28.46%) and then fair (18.96%) oral hygiene status subjects (Table 3).

**Table 3:** Representing the correlation of education and oral hygiene status of the study subjects HS: highly significant; N: number

Education	Frequency/percentage			Chi-square (χ^2^)	P-value	Significance
	Good (N = 455)	Fair (N = 156)	Poor (N = 189)			
Illiterate (40)	2/5	30/75	8/20	143.71	<0.000	HS
Class I-VIII (23)	18/78.26	2/8.7	3/13.04			
Class IX-XII (139)	120/86.34	10/7.19	9/6.47			
Graduate (580)	305/52.58	110/18.96	165/28.46			
Post-graduate (18)	10/55.54	4/22.23	4/22.23			

When the correlation of the frequency of brushing with that of education was done, highly statistically significant results were obtained. On evaluating proportions, about 88.5% of them brushed once a day, and only 8.87% of them brushed twice a day (Table 4).

**Table 4:** Representing the correlation between frequency of brushing and the education status of the study subjects N: number; HS: highly significant

Education	Frequency/percentage			Chi-square (χ^2^)	P-value	Significance
	Occasional (N = 21)	Once a day (N = 708)	Twice a day (N = 71)			
Illiterate (40)	7/17.5	30/75	3/7.5	58.60	<0.000*	HS
Class I-VIII (23)	2/8.70	18/78.26	3/13.04			
Class IX-XII (139)	5/3.60	130/93.50	4/2.9			
Graduate (580)	5/0.87	515/88.80	60/10.33			
Post-graduate (18)	2/11.12	15/83.34	1/5.54			
Total	2.63	88.5	8.87			

On evaluating the descriptive statistics of the study subjects, 88.37% of them used toothbrushes with a cleansing agent in the form of powder or paste, while 4.37% used neem sticks to clean their teeth; 53.75% used a combination of horizontal, vertical, and circular motions for cleaning their teeth. None was well aware of the effects of proper brushing techniques; 19.5% were well aware of the fact of changing their toothbrush after a regular interval of 3-6 months, while around 80.12% used the same toothbrush for more than six months or until fraying of the bristles was quite evident. When patients were asked about their tongue-cleaning habits, 79.5% of the subjects never cleaned their tongue, while 8.62% of them cleaned it using the bristles of the toothbrush only. A small population of around 0.75% only rinsed their mouth with water after eating, while 64.25% never did the same. Out of the total population, 53.25% of them have never ever used any mouthwash, and 6.25% did not use any interdental aid for maintaining their oral hygiene. The majority of the population of the study were involved in chewing paan, smoking, and the use of tobacco, thus compromising their periodontal and dental health (Table 5).

**Table 5:** Representing the descriptive analysis of the oral hygiene status of the subjects SD: standard deviation

Questions	Options available	Frequency/percentage	Mean ± SD
By what method do you clean your teeth?	Neem stick	35/4.37	2.84 ± 0.471
	Finger + toothpaste/powder	58/7.26	
	Toothbrush + toothpaste/powder	707/88.37	
	Any other	-	
What technique do you use for brushing?	Horizontal	250/31.25	2.85 ± 1.32
	Vertical	25/3.12	
	Circular	120/15	
	Combination	430/53.75	
How often do you change your brush?	Before three months	3/0.37	2.79 ± 0.411
	Between three and six months	156/19.5	
	More than six months	641/80.12	
Do you clean your tongue?	Daily	69/8.62	2.70 ± 0.616
	Occasionally	95/11.88	
	Never	636/79.5	
Do you rinse your mouth with plain water after eating?	Always	6/0.75	2.63 ± 0.497
	Sometimes	280/35	
	Never	514/64.25	
Have you ever used or do you use any mouthwash for oral hygiene?	Never	426/53.25	1.47 ± 0.499
	Occasionally	374/46.75	
	Regular	-	
Which of the below-mentioned interdental aids have you used?	Floss	-	3.06 ± 0.242
	Interdental brush	-	
	Toothpick	750/93.75	
	None	50/6.25	
Do you practice any of the deleterious habits?	Smoking	453/56.62	1.80 ± 0.951
	Tobacco	47/5.87	
	Chewing paan	300/37.5	
	None	-	

A comparative relationship with exceptionally high statistically significant results was obtained, between the time spend on brushing and education. Of the total population, 96.25% spends less than three minutes for brushing, and only 0.62% brushed more than three minutes, making a negligible amount (Table 6).

**Table 6:** Representing the correlation of education with the time spend on brushing HS: highly significant

Education	Frequency/percentage			Chi-square (χ^2^)	P-value	Significances
	<3 minutes (770)	1-3 minutes (25)	>3 minutes (5)			
Illiterate (40)	38	1	1	410.41	<0.000	HS
Class I-VIII (23)	21	1	1			
Class IX-XII (139)	136	2	1			
Graduate (580)	573	6	1			
Post-graduate (18)	2	15	1			
Total	96.25	3.12	0.62			

On evaluating the descriptive analysis regarding the awareness of periodontal and systemic diseases among the study subjects, 78.5% of the total population was aware of the term "pyorrhea" used for the gingival disease, and a very few (1.5%) were acquainted with the term periodontitis and gingivitis (20%); 85% knew about plaque and bacterial deposits as the main cause of gingival diseases, while 15% had no idea about the same. A large number of population were aware of the symptoms relating to gingival disease.

Perio-systemic interrelationship was the main question to be analyzed where only 3.25% knew about the relationship of bad oral hygiene and risk of heart attack and 1.87% with that of diabetes. Around 95% of the total population had no idea about this interrelationship (Table 7).

**Table 7:** Representing the descriptive analysis of the awareness regarding periodontal disease and perio-systemic interlink of study subjects SD: standard deviation

Questions	Options available	Frequency/percentage	Mean ± SD
What name or term of "gum disease" are you aware of?	Gingivitis	160/20	1.81 ± 0.425
	Pyorrhea	628/78.5	
	Periodontitis	12/1.5	
	None	-	
What do you think is the primary cause of gum disease?	Plaque/bacterial deposits	680/85	1.3 ± 0.71
	Food deposits	-	
	No idea	120/15	
Are you aware of any of the symptoms of gum disease?	Bleeding from the gums	709/88.62	
	Redness of the gums	25/3.12	
	Swelling of the gums	156/19.5	
	Bad breath	632/79	
	Mobility of the teeth	250/31.25	
	None	-	
Which perio-systemic interlinks are you aware of?	Bad oral hygiene and risk of heart attack	26/3.25	3.86 ± 0.59
	Bad oral hygiene and worsening of diabetes	15/1.87	
	Bad oral hygiene and risk of preterm birth	-	
	No idea	759/94.88	
How often do you visit a dentist?	Need based	354/44.25	2.13 ± 1.02
	Once in six months	2/0.25	
	Once in a year	424/53	
	My first visit	20/2.5	

On comparing the awareness of educated people about the perio-systemic interrelationship, statistically nonsignificant results were obtained (Table 8).

**Table 8:** Representing the correlation of education with the awareness of perio-systemic interrelationship NS: not significant

Education	Frequency/percentage				Chi-square (χ^2^)	P-value	Significance
	Heart attack (26)	Diabetes (15)	Preterm labor	No idea (759)			
Illiterate (40)	-	-	-	40	0.073	0.786	NS
Class I-VIII (23)	-	-	-	23			
Class IX-XII (139)	-	-	-	139			
Graduate (580)	15/57.69	8/53.33	-	557			
Post-graduate (18)	11/42.30	7/46.67	-	-			
Total	3.25	1.87	-	94.88			

## Discussion

Though an integral part of general health, oral health has always been neglected by one and all. People usually underestimate the consequences of bad oral health, which in turn is known for leading to bigger problems that later become difficult to treat. Periodontal disease forms one of the major risk factors for the development of various systemic conditions such as diabetes, osteoporosis, adverse pregnancy outcomes, and cardiovascular diseases, a topic that has been highly researched and debated [6]. The preponderance of the population is unaware of this relationship, although most evidence in this regard is consistently supportive. Many of the diseases showing oral signs and symptoms as their first appearance still remain undiagnosed or untreated mainly due to a lack of this awareness. Hence, many more studies need to be advocated by physicians and dentists to clear the fact and spread much awareness among the general population.

A large number of studies have been carried out in the past to assess the knowledge and attitudes of the general population about oral health; in any case, there is yet a deficiency of literature where oral hygiene practices and the awareness of periodontal systemic health interrelationship are assessed district- or region-wise. Thus, the present study was conducted with the main aim of evaluating the awareness of periodontal and systemic interrelationship with oral hygiene practices among the general population of Ranchi visiting the hospital. A total of 800 subjects, both males (68.75%) and females (31.25%) who were fulfilling the inclusion criteria, were selected for the study. The mean age of the patients was 37.61 ± 12.67, with a maximum number (N = 325) in the age group of 18-30 years. Out of 800 subjects, only 2.25% of them were post-graduates (N = 18), and 72.5% (N = 580) were graduates; 5% (N = 40) were illiterate with a mean and standard deviation of 3.643 ± 0.784.

The results of our study showed that the awareness and maintenance of oral hygiene were higher in females (61.2%) when compared with males (54.91%) with a maximum of 56.87% having fair oral hygiene status. This was in accordance with the study conducted by Hemalatha et al. [7] and Gupta et al. [8] where they found that females had more awareness regarding oral hygiene when compared with males. When the level of oral hygiene index was compared with that of the educational status of the study subject, a highly statistically significant result (p < 0.000) was obtained with a chi-square value of 143.71. The results of our study showed fair oral hygiene status in the majority of the population (N = 305) who were post-graduates, followed by graduates (N = 18.96) in the same category. This was in accordance with the study conducted by Vandana and Reddy, who found that around 73.9% of the population was in the fair category [9]. This reveals the results of the higher-educated group maintaining their oral hygiene status to a level of good (55.54%) when compared to illiterates, which were in turn in accordance with the study conducted by Hemalatha et al. [7].

With reference to oral hygiene practices, a greater number of patients used toothbrushes along with powder or paste (88.37%) for cleaning their teeth, with similar results found by Younus and Qureshi [10] and Ali et al. [11]. It was found that the use of neem sticks and finger was higher in illiterate people [10,11]. In our study, 96.25% of the subjects brushed for less than three minutes, with a maximum population of them being graduates (N = 573); 3.12% of them took 1-3 minutes in cleaning their teeth with the majority (N = 15) of them being post-graduates, and only 0.62% of them brushed for more than three minutes with equal frequency among all the education classes. Brushing for 2-3 minutes is considered good for maintaining oral hygiene [12]. Thirty-eight out of 40 illiterate patients brushed for less than three minutes, thus making education an important factor in spreading awareness about maintaining oral hygiene. A highly statistically significant correlation was found between education and the maintenance of oral hygiene status with increasing the time of brushing.

The technique followed by the majority of the population for brushing was a combination of horizontal, circular, and vertical motion. No specific knowledge about the brushing techniques was followed by the people. Around 80.12% of them change their toothbrush after six months, while the rest discarded their toothbrush either before three months or between three- and six-month intervals. Tongue-cleaning methods, the use of interdental aids, and cleaning of the mouth using water or using mouthwash were minimal among the study population; 79.5% of them had never used a tongue cleaner, 64.25% never rinsed their mouth after eating food, 53.25% never used mouthwash, and 93.75% used toothpicks as an interdental cleaning aid. These parameters were inconsistent with the studies conducted by Jamjoom [13] and Jain et al. [6]. Educating and motivating the subjects along with the general population were an urgent need for making an efficient and emphasized use of these oral hygiene aids and oral health care measures [8].

When evaluating the awareness of periodontal and systemic health relationship, an awareness system was evaluated on the basis of the knowledge of the subjects in regard to the relation of periodontal diseases with that of the heart, diabetes, and preterm labor. It was found that only 3.25% and 1.87% were aware of this interrelationship. None knew about the relationship between preterm labor and periodontal health. This made a poor awareness association of subjects with periodontal diseases, which was assessed using the answer with a maximal response. The results of our study were similar to those conducted by Kapoor et al. [14], Gupta et al. [8], and Bhatia et al. [15], who surveyed the local population of Punjab and found that people were unaware of the relationship. Of the total population, 78.5% was aware of the term "pyorrhea," which was the commonest of all. Around 1.5% knew about periodontitis, and 20% had an idea about the term gingivitis.

From the results accomplished in our study, it was quite evident that study subjects had little awareness of the interrelationship. Hence, due motivation, the awareness of periodontal maintenance, and the achievement of healthy oral hygiene are of utmost importance. Patients with systemic diseases must be easily distinguished from the general population and constrained to have strict follow-up visits in dental clinics or hospitals. Not only the patients visiting the dental setups but also the general population should be made aware of the periodontal and systemic interrelationship and the impact of maintaining oral hygiene.

## Conclusions

Within the limitations of our study, it can be concluded that a space for oral hygiene practices and self-care standards among the general population of Ranchi, Jharkhand, should be created. Furthermore, there is a lack of appropriate oral health awareness, specifically regarding the perio-systemic relationship, even among the educated, which should not be overlooked. Hence, there is an urgent need to educate and spread awareness along with the knowledge of proper oral and dental care to make an individual healthy.

## References

[1] Newman HN (1996). Focal infection. J Dent Res.

[2] Mattila KJ, Nieminen MS, Valtonen VV (1989). Association between dental health and acute myocardial infarction. BMJ.

[3] Loesche WJ, Grossman NS (2001). Periodontal disease as a specific, albeit chronic, infection: diagnosis and treatment. Clin Microbiol Rev.

[4] Umeizudike KA, Iwuala SO, Ozoh OB, Ekekezie OO, Umeizudike TI (2015). Periodontal systemic interaction: perception, attitudes and practices among medical doctors in Nigeria. J West Afr Coll Surg.

[5] Scannapieco FA (1998). Position paper of the American Academy of Periodontology: periodontal disease as a potential risk factor for systemic diseases. J Periodontol.

[6] Jain N, Mitra D, Ashok KP, Dundappa J, Soni S, Ahmed S (2012). Oral hygiene-awareness and practice among patients attending OPD at Vyas Dental College and Hospital, Jodhpur. J Indian Soc Periodontol.

[7] Hemalatha DM, Melath A, Feroz M, Subair K, Mohandas A, Chandran N (2020). A survey on the awareness of interrelationship of periodontal disease and systemic health among Mahe population. J Indian Soc Periodontol.

[8] Gupta V, Singh AK, Gupta B (2016). Assessment of oral hygiene practices and awareness of periodontal-systemic health interrelationship amongst the local population of Kanpur region - a cross sectional study. J Oral Health Community Dent.

[9] Vandana KL, Reddy MS (2007). Assessment of periodontal status in dental fluorosis subjects using community periodontal index of treatment needs. Indian J Dent Res.

[10] Younus A, Qureshi A (2016). Tooth brush changing frequency and associated socio-demographic and oral hygiene factors among residents of Karachi. J Dent Oral Hyg.

[11] Ali NS, Khan M, Butt M, Riaz S (2012). Implications of practices and perception on oral hygiene in patients attending a tertiary care hospital. J Pak Dent Assoc.

[12] Ganss C, Schlueter N, Preiss S, Klimek J (2009). Tooth brushing habits in uninstructed adults--frequency, technique, duration and force. Clin Oral Investig.

[13] Jamjoom HM (2001). Preventive oral health knowledge and practice in Jeddah, Saudi Arabia. J King Abdulaziz Univ Med Sci.

[14] Kapoor D, Gill S, Singh A, Kaur I, Kapoor P (2014). Oral hygiene awareness and practice amongst patients visiting the Department of Periodontology at a Dental College and Hospital in North India. Indian J Dent.

[15] Bhatia A, Singh M, Bains S (2013). To assess knowledge and awareness of north Indian population towards periodontal therapy and oral-systemic disease link: a cross-sectional survey. J Interdiscip Dent.

